# The Emerging Roles of circSMARCA5 in Cancer

**DOI:** 10.1155/2022/3015818

**Published:** 2022-06-07

**Authors:** Haoyu Qin, Renhua Wan

**Affiliations:** ^1^Medical School of Nanchang University, Nanchang University, Nanchang, China; ^2^Department of General Surgery, The First Affiliated Hospital of Nanchang University, Nanchang University, Nanchang, China

## Abstract

Circular RNAs have a unique covalent closed-loop structure, which is mainly formed by the reverse splicing of exons from a precursor mRNA. With the development of key technologies such as high-throughput sequencing and the advancement of bioinformatics in recent years, our understanding of circular RNAs has become increasingly more detailed, and their abnormal expression in a variety of cancers has attracted increasing attention. Studies have shown that circSNARCA5 not only plays a crucial role in the occurrence and development of cancer but may also serve as a reliable indicator for tumor screening or a good marker for evaluating cancer prognosis. Nevertheless, there are no reviews focusing on the relationship between circSMARCA5 and cancer. Therefore, we will first explain the main biological characteristics of circSMARCA5, such as biogenesis and biological effects. Then, the focus will be on its role and significance in cancer. Finally, we will summarize the known information on circSMARCA5 in cancer and discuss future research prospects.

## 1. Introduction

Circular RNAs (circRNAs) are continuous, single-stranded endogenous RNAs characterized by a closed circular structure without a 5′-terminal cap or a 3′-end poly (A) tail [1, 2]. In 1976, Sanger [3] first observed covalently closed circular RNA molecules in RNA viruses. CircSMARCA5 were only reported by chance after this discovery and were commonly considered to merely be an intermediate of intron lasso detachment or RNA splicing errors. Few thought circRNAs might have important biological roles, and they were consequently largely ignored. With the development of high-throughput sequencing and bioinformatics, circular RNAs gradually entered the focus of researchers. At the same time, an increasing number of studies showed that circular RNAs are widely expressed in human cells, with expression levels of some circRNAs, which is reaching more than ten times that of homologous mRNAs. There are many different circular RNAs in the human body, which are closely related to the occurrence, development, and prognosis of diseases [4]. About one-third of circRNAs are relatively conserved among species and can be dynamically expressed at different developmental stages in different tissues. CircSMARCA5 can be divided into exon circSMARCA5 (EcircRNAs), intron circSMARCA5 (ciRNAs), and exon-intron circSMARCA5 (EIciRNAs). Generally speaking, most EcircRNAs are distributed in the cytoplasm and have the potential to bind RNA-binding proteins (RBPs) and other RNAs, while ciRNAs and EIciRNAs are mainly found in the nucleus, which may make them potentially useful in transcription or regulation. There are currently three models for the generation of circRNA, including intron pair-driven cyclization, lasso-driven cyclization, and RBP-driven cyclization. Due to the unique structure of circular RNAs, they cannot be degraded by exonucleases and are more stable, with much longer half-lives than corresponding linear RNAs. Crucially, their increased stability makes them easier to detect as biomarkers [5, 6].

CircSMARCA5 plays a role mainly through their interaction with other RNAs [7–9]. CircSMARCA5 usually have many binding sites for miRNAs and regulate the functions of miRNAs by acting as a sponge [4]. For example, CircRNA CIRS-7 has many binding sites for miR-7, which reduces the biological activity of miR-7 due to sponging. CircRNA can also be combined with corresponding target mRNAs to regulate gene expression. For example, CDR1as modulates the activity of target miRNAs by binding in trans to 3′UTRs. The role of circRNA does not stop there. Some RNAs may interact with circRNAs in the nucleus, which may have the effect of promoting transcription and enhancing gene expression. CircSMARCA5 can also compete for the splicing sites of pre-mRNAs, thereby reducing mRNA production. Interestingly, internal ribosome entry sequences (IRES) can drive ribosomes or initiation factors to combine with certain circRNA, which means that circRNAs can also be translated. Finally, circRNA can also be combined with RBPs and enhance protein-protein interactions [4].

Next, we will combine the literature to describe the characteristics and potential mechanisms of circSMARCA5 in a variety of cancers, review the results and ideas of multiple studies, provide a basis for further research, and actively explore the significance of circSMARCA5 for cancer treatment and diagnosis.

## 2. Biological Characteristics of circSMARCA5

In recent years, numerous studies reported that circRNAs have different degrees of influence on the occurrence and progression of diseases through various mechanisms. In cancer, circRNAs affect cell proliferation, differentiation, and apoptosis, thereby influencing tumor formation and progression. Therefore, the research on the role of circRNAs in cancer is particularly important. SMARCA5 (also called SNF2H) is a member of the ISWI family of chromatin remodelers. It regulates chromosome remodeling through a variety of mechanisms [10]. Like other circRNAs, circSMARCA5 can sponge miRNAs to inhibit cell proliferation and promote apoptosis. Interestingly, the expression of circSMARCA5 may also enhance the susceptibility of cells to chemotherapy, so it may provide a new way to treat chemotherapy-resistant cancers [11–13]. Many studies have shown that the expression level of this circRNA has a certain relationship with clinical characteristics of many cancers. In some cases, it can be detected at a higher level of expression in the early stages of cancer. In liver diseases, liver cancer, hepatitis, and cirrhosis can be clearly distinguished by the expression level of circSMARCA5, which can improve the sensitivity of cancer detection, promoting early diagnosis. Thus, research on circSMARCA5 is helpful for the secondary prevention of cancer [11–15].

### 2.1. Biogenesis of circSMARCA5

**Figure 1:**
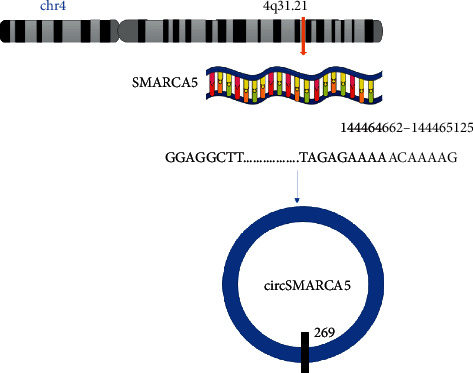
Schematic diagram illustrating the biogenesis of circSMARCA5. Exons 15 and 16 of 4q31.21 on chr4 are cyclized to form circSMARCA5 (blue arrow).

**Figure 2:**
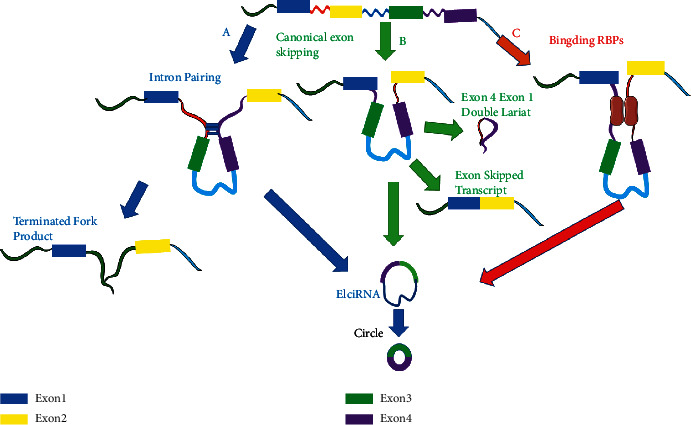
Models for circRNA generation A. Various secondary structures of RNA lead to spatially favorable positions for circularization B. The lasso structure formed by skipping exons to generate a circRNA. C. After a RBP binds to RNA, it acts as a bridge to bring RNA strands closer to each other to promote circularization.

In general, pre-mRNAs formed after transcription can be converted into circRNAs. The formation of circRNAs mainly depends on the cutting method, and theoretically, it is controlled by cis-regulatory elements and trans-acting factors. Although the specific mechanism of reverse shearing is not clear, it is certain that reverse shearing requires special tools, and its efficiency is lower than that of standard shearing, resulting in a lower steady-state level of circRNAs compared to their linear counterparts. The efficiency of reverse shearing mainly depends on the shearing sites around the exons. The above-mentioned circSMARCA5 (hsa_circ_0001445) is produced from exons 15 and 16 of the SMARCA5 gene located on chromosome 4q31.21 and has a short length of only 269 nucleotides (Figure 1) [10, 16]. CircSMARCA5 is widely expressed and exists in the cytoplasm and internal environment, so it plays an important role in the human body [14]. Oligo dT primer amplification experiments showed that circSMARCA5 does not have a poly(A) tail [17]. RNase cleavage experiments further confirmed that circSMARCA5 is a closed loop and is more stable than the corresponding mRNA [17]. It was reported that circSMARCA5 requires long inverted repeats to perform its biological functions. Some studies have found an evolutionarily conserved 132-nucleotide inverted repeat sequence in the pre-mRNA of SMARCA5 [18]. These results give us a deeper understanding of the biogenesis of circSMARCA5 (Figure 2).

### 2.2. The Biological Function of circSMARCA5

circSMARCA5 is expressed in a variety of cells [14]. Among them, it is highly enriched in the myocardium, and its levels are correlated with the pathological state of the coronary artery wall. Therefore, researchers have pointed out that circSMARCA5 can be used as an effective biomarker of coronary atherosclerosis [19]. Like many other circRNAs, circSMARCA5 acts as an RNA sponge to regulate the levels of other miRNAs [13, 16, 20–25]. For example, circSMARCA5 can reduce the inflammatory response of ox-LDL-induced HUVECs and inhibit their apoptosis by sponging miRNA-640. Additionally, circSMARCA5 can also bind to RBPs to regulate their level or biological activity, thereby exerting biological effects [26, 27]. CircSMARCA5 can be guided to its parental gene locus, thereby forming an R-loop, terminating its gene transcription, and truncating circSMARCA5 protein. Therefore, the expression of the SMARCA5 gene decreases [28]. Thus, the study of the biological role of circSMARCA5 in cancer is particularly important.

### 2.3. Expression of circSMARCA5 in Cancers

circSMARCA5 expression exhibits abnormal fluctuations in many cancers. Its expression is reduced in most cancers, such as CRC, MM, ependymoma, NSCLC, intrahepatic cholangiocarcinoma (ICC), HCC, and glioblastoma multiforme (GBM) (Table 1) [11–14, 18, 20–22, 30]. In these cases, circSMARCA5 mostly plays a role in inhibiting the occurrence, development, and metastasis of cancer. However, there are also studies showing that the expression of circSMARCA5 is decreased in CC, even if these results remain controversial [16, 23, 27]. The expression level circSMARCA5 in prostate cancer (PCa) is also different from other cancers, since it is highly expressed in PCa as an oncogenic factor [24, 31, 32].

## 3. Dual Roles of circSMARCA5 in Different Cancers

### 3.1. Colorectal Cancer

Colorectal cancer is the most common malignant tumor that originates in the colon and rectum, and it remains a leading cause of cancer-related deaths worldwide. Although recent studies have shown that the incidence of colorectal cancer is decreasing in some regions [33, 34], overall, the prognosis of colorectal cancer is still very poor, and there is an urgent need for effective examinations for early detection, as well as treatments that can improve the prognosis [35].

Studies have shown that overexpression of circSMRCA5 can inhibit the proliferation, metastasis, and invasion of colorectal cancer, and these effects can be achieved through different modes of action [21, 36]. First of all, circSMARCA5 can promote the expression of ARID4B by sponging miR-39-3p. ARID4B was reported to have a tumor suppressor effect in other cancers, such as prostate cancer and leukemia, so circSMARCA5 may indirectly play a role in suppressing cancer through this pathway [21]. In another study, circSMARCA5 was found to bind miR-552, resulting in a decrease in the expression of various cell proliferation factors such as p53 and p21, and eventually interrupting the Wnt and YAP1 pathways, thereby suppressing cancer [36].

### 3.2. Liver Cancer

Liver cancer is one of the cancers with the highest mortality rate in the world [37]. Although the therapy of liver cancer is constantly improving, the five-year survival rate is still low [38]. Due to the insidious nature of the disease, most patients are diagnosed at an advanced stage, which is an important reason for the high mortality. Therefore, finding safe, effective, simple, and inexpensive early screening and diagnosis indicators is a very important research direction for liver cancer.

Studies have pointed out that circSMARCA5 can specifically sponge miR-17-3p and miR-181b-5p. Furthermore, TIMP3 is a common target of miR-173p and miR-181b-5p, which can significantly inhibit the expression of TIMP3. Conversely, circSMARCA5 can increase the expression of TIMP3 by sponging miR-17-3p and miR-181b-5p [25]. TIMP3 has been proven to act as a tumor suppressor that can effectively inhibit the proliferation and metastasis of HCC [39–42]. Studies have also shown that circSMARCA5 can inhibit the expression of matrix metalloproteinases (MMPs) [43], which are key factors that promote tumor invasion and metastasis [14]. Accordingly, circSMARCA5 expression is not only correlated with the size of HCC, TMN stage, and degree of differentiation [25, 43], but also has a significant relationship with the degree of microvascular infiltration, OS, and RFS [25]. The specificity and sensitivity of alpha-fetoprotein for liver cancer has made it an important indicator for liver cancer screening and diagnosis, but only 15–30% of liver cancer patients have significant changes in AFP in the early stage. This also caused difficulties for the early diagnosis of liver cancer. However, circSMARCA5 research has yielded encouraging results. The stability of circRNA in plasma makes it an effective potential screening and diagnostic indicator. Studies have pointed out that the level of circSMARCA5 can effectively distinguish patients with liver cancer hepatitis and liver fibrosis rom healthy controls, and it is also meaningful in patients with hepatitis and cirrhosis with AFP < 200 ng/ml. Therefore, it may be used as a screening indicator for patients with hepatitis or cirrhosis with low AFP levels [14]. For developing countries such as China, which are prone to liver cancer, liver cancer is already very serious when patients are admitted to the hospital due to the difficulty of screening. This is a serious burden on the country, and this burden will be greatly reduced if effective, convenient, and inexpensive screening indicators can be found. CircSMARCA5 can not only screen for liver cancer but also has the ability to distinguish between liver fibrosis and hepatitis, and may be used as a screening indicator for cancer together with alpha-fetoprotein.

### 3.3. Intrahepatic Cholangiocarcinoma

Intrahepatic cholangiocarcinoma is a primary malignant tumor of the liver whose incidence is second only to hepatocellular carcinoma, and has been increasing over the past 20 years [12, 44, 45]. The five-year survival rate is poor, and there is no effective other than surgical resection. At present, the long-term survival rate of patients is poor due to factors such as relapse and chemotherapy resistance.

Studies revealed that the expression of circSMARCA5 in cancer tissues was significantly decreased relative to the surrounding tissues, and the level of circSMARCA5 was negatively correlated with the ECOG function score, TNM staging, and CA199 abnormal state, indicating that it can be used as an independent prognostic marker. It was also found that circSMARCA5 can not only inhibit the invasion and proliferation of cancer cells by itself but also increase their chemosensitivity to cisplatin/gemcitabine. This may be one of the factors why circSMARCA5 expression predicts a good prognosis in a variety of cancers [46].

### 3.4. Non-Small-Cell Lung Cancer

Lung cancer is one of the leading causes of cancer-related deaths worldwide. The two-year relative survival rate of patients with non-small-cell lung cancer has increased from 34% in 2009–2010 to 42% in 2015–2016 [37]. Although the treatment technology and diagnostic methods of non-small-cell lung cancer are constantly improving, its five-year survival rate is still very low. We need to further understand its biological basis and underlying molecular mechanisms, which will provide effective early biomarkers and prognostic evaluation indicators [11].

Studies have shown that circSMARCA5 can not only sponge miR-19b-3p, thereby increasing the expression of HOXA9 [22], but also exert a tumor suppressor effect by regulating the miR-670-5p/RBM24 axis [13]. In general, circSMARCA5 has a significant relationship with DFS, tumor size, LYN metastasis, TNM stage, and CEA of lung cancer and can also be used as an independent predictor of lung cancer prognosis [11, 13, 22].

### 3.5. Cervical Cancer

Although cervical cancer can be easily screened, more than 4000 women lost their lives due to this malignancy in 2018 alone. Among women aged 20 to 39, it is still the second leading cause of cancer-related deaths [37]. Although not all causes of cervical cancer are known, human papillomavirus infection is considered to be the main risk factor [47].

Studies have shown that SND1 is an RBP that binds to circSMARCA5. SND1 has been proven in other studies to promote the proliferation and invasion of a variety of cancers [48–50], and it can also bind to YWHAB. Although YWHAB contributes to the development of cancer [51], its overexpression has a tumor suppressor effect. Accordingly, circSMARCA5 can inhibit the interaction of SND1 and YWHAB, and can also reduce the level of SND1, thereby exerting a tumor suppressor effect [52]. There are also studies, indicating that circSMARCA5 can sponge mi-620 and inhibit the proliferation, migration, and invasion of CC cells [16]. However, there are also findings that circSMARCA5 is upregulated in cervical cancer cells. It also acts as a sponge for miR-432, thereby inhibiting its binding to the 3′UTR of EGFR, which has the effect of promoting cancer cell proliferation and invasion [23]. Additionally, circSMARCA5 can also upregulate the ERK1/2 signaling pathway, which induces the proliferation and invasion of cervical cancer [27]. Cervical cancer is also a big problem for countries in East, South, or West Africa. CircSMARCA5 may become an important target for cervical cancer treatment and early screening. For these relatively underdeveloped but large population areas, effective, convenient, and inexpensive indicators are particularly important.

### 3.6. Bladder Cancer

Bladder cancer is a malignant tumor that occurs in the bladder mucosa. It is the most common malignant tumor of the urinary system, with 400,000 new cases each year [53]. It was reported that the expression of circSMARCA5 is significantly related to the lesion number, size, stage, lymph node metastasis, and pathological types of bladder cancer. Generally speaking, the increase of circSMARCA5 can inhibit the occurrence and development of cancer [54].

### 3.7. Multiple Myeloma

Multiple myeloma is a malignant tumor caused by the abnormal proliferation of plasma cells, often accompanied by multiple osteolytic damage, hypercalcemia, anemia, and kidney damage [20]. Although the treatment of MM continues to improve, it is prone to recurrence, and a lack of complete remission (CR) after recurrence is the main reasons for the low survival rate. Therefore, new targets are needed to improve the prognosis of patients [20]. Studies have found that circSMARCA5 in myeloma often indicates a lower ISS stage in which it is easier to achieve CR. In this cancer, circSMARCA5 may exert its tumor suppressor effect by sponging miR-767-5p [55].

### 3.8. Glioblastoma Multiforme

Glioblastoma multiforme is the most common malignant primary brain tumor in adults. It is highly aggressive, and there is still no effective treatment method. The median survival time following diagnosis is approximately 16 months [55–58]. GBM is a grade IV glioma, and its main characteristics are recalcitrance to treatment and aggressive spread [59, 60]. Since the WHO included biomarkers in the central nervous system tumor classification in 2016, research on the molecular mechanisms of GBM has become increasingly active [60]. Compared with linear RNAs, circRNAs are more stable and more abundant in neural cells, which make them an important target for central nervous system tumors [61, 62].

The role of circSMARCA5 in GBM is closely related to serine and arginine-rich splicing factor 1 (SRSF1) [18, 30, 63, 64]. It plays a role in suppressing cancer indirectly through a variety of pathways. First, SRSF1 is tethered by circSMARCA5 as an RNA-binding protein. GAUGAA may be a more critical RNA sequence, and studies have shown that SRSF1 is more likely to bind to GA-rich RNAs [18]. SRSF1 was confirmed to be a cancer promoter that can effectively promote the proliferation and invasion of cancer cells. It can induce SRSF3 pre-RNA to skip exon 4, thereby controlling the expression of its subtypes. SRSF1 controls the splicing mode of SRSF3, which in turn can regulate the expression of PTBP1, while the overexpression of PTBP1 can also promote the migration of GBM cells [63, 65]. VEGFA can increase the permeability of blood vessels and enhance the dissemination of certain proteins, which are necessary for the sprouting of endothelial cell tubes to generate blood vessels. Tumors, especially solid tumors, are basically hypoxic, so angiogenesis is very important for tumor growth [9, 66]. The pre-RNA of VEGFA can be selectively cut into pro-angiogenic isoforms (VEGF-Axxxa) or anti-angiogenic isoforms (VEGF-Axxxb), and these depend on the amount of SRSF1 binding the proximal splicing site (PSS) within the eighth exon of VEGFA pre-mRNA, which will increase the retention rate of the full-length eighth exon and promote the synthesis of VEGF-Axxxa. CircSMARCA5 reduces the level of VEGF-Axxxa by downregulating SRSF1, thereby inhibiting tumor proliferation and invasion [64, 66]. In general, the expression of circSMARCA5 in GBM is correlated with a better prognosis [18, 30, 63, 64]. CircSMARCA5 is considered a new potential biomarker, while increasing circSMARCA5 expression in cancer cells represents a new direction for cancer treatment.

### 3.9. Prostate Cancer

Prostate cancer (PCA) is one of the most common cancers affecting men in the world. The androgen receptor pathway is crucial for the occurrence and development of PCa, and genetic factors are considered to be dominant [67, 68].

**Figure 3:**
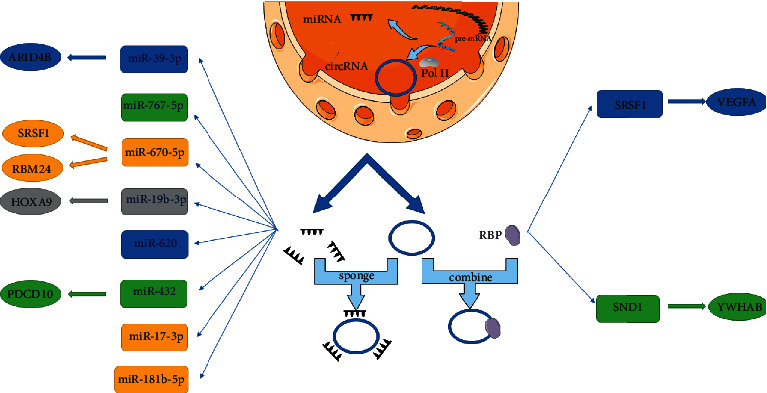
Schematic diagram of the biological functions of circSMARCA5. CircSMARCA5 is generated in the nucleus and then enters the cytoplasm, where it can sponge miRNAs and exert its own effects through downstream factors. It can also bind to RNA-binding proteins to exert diverse biological effects.

Relevant studies indicate that the level of circSMARCA5 is increased in cancer tissues [24, 32], which can promote the entry of cells from the G1 phase into the S phase, thereby promoting cancer cell proliferation and migration [32]. In PCa, circSMARCA5 mainly sponges miR-432 to indirectly increase the level of PDCD10, thus exerting a cancer-promoting effect [24].  Figure 3 shows the Schematic diagram of the biological functions of circSMARCA5.

## 4. Conclusions

The important roles of circRNAs in different cancers have received increasing attention. Due to their unique properties and functions, circRNAs are of great significance for the diagnosis, treatment, and prognosis of cancer. For mouse models, antisense of circSMARCA5, as a traditional and effective method, may become an important model to study its relationship with cancer in the future. Many studies have shown that circRNAs participate in tumor growth and metastasis through many potential mechanisms. Different circRNAs have different pathways and play different roles. This article focuses on retrospective analysis and discussion of multiple studies on the roles of circSMARCA5 in cancer. Many studies have shown that the expression level of circSMARCA5 is significantly related to TNM stage, lymph node metastasis, and other cancer-related indicators [11–14]. In most cancers, the level of circSMARCA5 is low and mostly acts as a tumor suppressor that inhibits cell growth and promotes apoptosis. However, it was found that circSMARCA5 can also act as an oncogene in PCa. He may play a role through sponge miR-432 as a cancer-promoting factor. Interestingly, some studies have shown that the expression level of circSMARCA5 is lower in cervical cancer than in normal tissues, while other studies found that it is elevated and can also promote cancer growth by sponging miR-432. Although the relevant research is progressing, there are still many potential mechanisms that have not been clarified. For example, the mechanism through which circSMARCA5 can increase the sensitivity of cancer cells to chemotherapy is still not fully understood. Many studies suggest that the expression level of circSMARCA5 has a high correlation with the patient's OS, PFS, and DFS [11, 12, 20, 22, 25, 30]. One study compared the expression levels of circSMARCA5 in hepatitis, liver cirrhosis, and hepatocellular carcinoma and found that the level of circSMARCA5 in the plasma is almost the same when there is no difference in AFP. These liver diseases are well distinguished, so circSMARCA5 may be used as a novel biomarker for the diagnosis of hepatocellular carcinoma [14]. We previously mentioned the unique structure of circRNAs, which are more stable in body fluids such as plasma and more easily detected than linear RNAs. Before this, RNA was rarely used as an indicator for routine cancer screening or detection due to the difficulty of extraction and preservation. Even if it could, it would be replaced by better detection methods due to the high cost. However, circSMARCA5 does not have the above difficulties, and its stable structure can exist more stably in plasma. Therefore, according to the current research, the level of circSMARCA5 can not only be used as a good biomarker for patient prognosis but also can make progress in cancer screening. In addition, we also need more comprehensive algorithms, such as monarch butterfly optimization (MBO), earthworm optimization algorithm (EWA), and elephant herding optimization (EHO) to study the connection between circSMARCA5 and other cancers, in order to better to exclude other factors interfering with the study. With further research, it is believed that more potential mechanisms will be discovered. Through new methods and unremitting efforts, circRNAs can play a more important role in oncology and enable simpler early diagnosis of many cancers, while also being potential therapeutic targets in many cases.

## Figures and Tables

**Table 1:** Expression and functions of circSMARCA5 in different cancers.

Cancer type	Expression	Functional roles	Related signaling pathways	References
Colorectal cancer	Downregulated	Proliferation, migration, invasion	miR-39-3p-ARID4B	[21]
Multiple myeloma	Downregulated	Proliferation, migration, invasion	miR-767-5p	[20]
Ependymoma	Downregulated	Proliferation, migration, invasion		[29]
Non-small-cell lung cancer	Downregulated	Proliferation, migration, invasion chemoresistance	miR-670-5p-RBM24 miR-670-5p-SRSF1 miR-19b-3p/HOXA9	[11, 13, 22]
Intrahepatic cholangiocarcinoma	Downregulated	Proliferation, migration, invasion chemoresistance		[12]
Hepatocellular carcinoma	Downregulated	Proliferation, migration, invasion	miR-17-3p miR-181b-5p	[14, 25]
Cervical cancer	Downregulated	Proliferation, migration, invasion	miR-620 SND1-YWHAB	[23, 27]
Cervical cancer	Upregulated	Proliferation, migration, invasion	miR-432-ERK1/2	[16]
Glioblastoma multiforme	Downregulated	Proliferation, migration, invasion	SRSF1-VEGFA	[18, 30]
Prostate cancer	Upregulated	Proliferation, migration, invasion	MiR-432-PDCD10	[24]

## Data Availability

The data used to support the findings of this study are available from the corresponding author upon request.
